# Widespread brain reorganization perturbs visuomotor coordination in early glaucoma

**DOI:** 10.1038/s41598-019-50793-x

**Published:** 2019-10-02

**Authors:** Vivek Trivedi, Ji Won Bang, Carlos Parra, Max K. Colbert, Caitlin O’Connell, Ahmel Arshad, Muneeb A. Faiq, Ian P. Conner, Mark S. Redfern, Gadi Wollstein, Joel S. Schuman, Rakie Cham, Kevin C. Chan

**Affiliations:** 1 Department of Ophthalmology, New York University (NYU) School of Medicine, NYU Langone Health, New York, NY USA; 2 Department of Bioengineering, Swanson School of Engineering, University of Pittsburgh, Pittsburgh, PA USA; 3 Department of Kinesiology, East Carolina University, Greenville, NC USA; 4 Department of Ophthalmology, School of Medicine, University of Pittsburgh, Pittsburgh, PA USA; 5 Department of Radiology, New York University (NYU) School of Medicine, NYU Langone Health, New York, NY USA; 6 Neuroscience Institute, New York University (NYU) School of Medicine, NYU Langone Health, New York, NY USA; 7 Center for Neural Science, Faculty of Arts and Science, New York University, New York, NY USA

**Keywords:** Diagnostic markers, Optic nerve diseases, Vision disorders

## Abstract

Glaucoma is the world’s leading cause of irreversible blindness, and falls are a major public health concern in glaucoma patients. Although recent evidence suggests the involvements of the brain toward advanced glaucoma stages, the early brain changes and their clinical and behavioral consequences remain poorly described. This study aims to determine how glaucoma may impair the brain structurally and functionally within and beyond the visual pathway in the early stages, and whether these changes can explain visuomotor impairments in glaucoma. Using multi-parametric magnetic resonance imaging, glaucoma patients presented compromised white matter integrity along the central visual pathway and around the supramarginal gyrus, as well as reduced functional connectivity between the supramarginal gyrus and the visual occipital and superior sensorimotor areas when compared to healthy controls. Furthermore, decreased functional connectivity between the supramarginal gyrus and the visual brain network may negatively impact postural control measured with dynamic posturography in glaucoma patients. Taken together, this study demonstrates that widespread structural and functional brain reorganization is taking place in areas associated with visuomotor coordination in early glaucoma. These results implicate an important central mechanism by which glaucoma patients may be susceptible to visual impairments and increased risk of falls.

## Introduction

Glaucoma is the world’s leading cause of irreversible blindness and is projected to affect 111.8 million people worldwide by 2040^1^. Glaucoma is defined by progressive retinal ganglion cell death, making the eye the primary focus for therapies and research. While intraocular pressure is currently the only modifiable risk factor for glaucoma, the disorder can continue to progress even at low intraocular pressures^2^. This suggests that other unexplored key factors are contributing to the disease onset and progression^3^. Over the past decade, seminal works from Calkins’ and Sponsel’s groups demonstrate that the central nervous system controls glaucomatous neurodegeneration in both humans and murine models in the early stages^4–8^. Recent neuroimaging research also demonstrates the occurrence of changes in a number of brain structures in glaucoma, both within and outside visual processing areas^9–17^. However, current literature on glaucoma neuroimaging remains sparse and incomplete, and is mostly limited to studies with small sample sizes and toward advanced disease stages^18^. In addition, the behavioral relevance of these findings remains unclear.

Alongside vision loss, falls are a major public health concern in glaucoma patients^19–21^. It has been reported that vision impairment, e.g. visual field loss, can reduce a patient’s ability to detect environmental hazards, causing trips and stumbles that can ultimately lead to injuries from falls^22,23^. Further, there is some evidence that patients with glaucoma may also have impaired ability to integrate multisensory inputs relevant for postural control, resulting in loss of balance and falls^24–26^. Inputs from the visual, vestibular and somatosensory systems are required for postural control^27^. When sensory information from one of these systems becomes compromised, individuals who are otherwise healthy are able to compensate for this loss by adjusting the contributions of the other sensory systems appropriately to maintain balance. This process is referred to as multisensory re-weighting or integration. Glaucoma patients have been found to have worse balance than healthy individuals when their sensory integration is challenged^28,29^, suggesting that poor multisensory integration may be another risk factor for compromised balance and fall risk in glaucoma patients. To date, the relationship between glaucomatous brain changes and postural control has not been investigated.

The purpose of this study is to identify structural and functional abnormalities in balance-related areas of the brain and determine whether these alterations are associated with postural control impairments in glaucoma patients. Understanding the multidimensional and complex mechanisms of falls in glaucoma patients is important to develop effective impairment-targeted falls prevention and rehabilitation therapies. Therefore, in this study, we use advanced neuroimaging and computerized dynamic posturography to assess brain and behavioral changes in glaucoma. We hypothesize that brain alterations in glaucoma are associated with multisensory integration dysfunction causing balance and gait impairments, which subsequently contributes to falls.

## Results and Discussion

In this observational study, 32 glaucoma subjects [age = 62.5 ± 1.5 (mean ± standard error); 40.6% male] and 10 healthy controls [age = 64.5 ± 2.9; 30.0% male] underwent clinical ophthalmic assessments including spectral-domain optical coherence tomography (OCT) and standard automated perimetry (SAP), followed by multi-parametric magnetic resonance imaging (MRI) including anatomical MRI, microstructural diffusion tensor MRI, and task-free functional MRI (tf-fMRI) (See Supplementary Information 1 for details). As shown in Table 1, there were significant differences in clinical ophthalmic assessments between glaucoma patients and healthy controls but not age or gender difference. For volumetric MRI measures, the optic nerve was significantly smaller in the glaucoma patients than healthy controls, while no apparent difference in grey matter volume was found in the primary or secondary visual cortex. Average peripapillary retinal nerve fiber layer (RNFL) thickness in OCT was significantly associated with visual field mean deviation (VF-MD) in SAP (r = 0.52, p < 0.001), whereas optic nerve volume was significantly associated with both VF-MD (r = 0.79, p < 0.001) and average RNFL thickness (r = 0.72, p < 0.001).

**Table 1:** Demographics, clinical ophthalmic assessments and volumetric MRI assessments of glaucoma and healthy control groups.

	Glaucoma (n = 32)	Healthy Control (n = 10)	P-values	Significance
Age (year)	62.5 ± 1.5	64.5 ± 2.9	0.527	ns
Sex	13M, 19F	3M, 7F	0.546	ns
Average RNFL thickness (µm) OD	74.5 ± 2.3	88.6 ± 3.5	0.011	*
Average RNFL thickness (µm) OS	74.6 ± 2.3	89.0 ± 3.1	0.010	*
Optic nerve head C/D OD	0.66 ± 0.03	0.46 ± 0.07	0.002	*
Optic nerve head C/D OS	0.68 ± 0.03	0.40 ± 0.08	0.001	*
Visual field mean deviation (dB) OD	−3.80 ± 1.09	−1.17 ± 0.29	0.189	ns
Visual field mean deviation (dB) OS	−4.37 ± 0.87	−0.64 ± 0.18	0.023	*
Optic nerve volume (mm^3^) OD	429.9 ± 87.8	534.7 ± 52.9	0.001	*
Optic nerve volume (mm^3^) OS	430.9 ± 85.3	534.2 ± 51.7	0.001	*
V1 grey matter volume (mm^3^)	8517.8 ± 1485.8	8302.8 ± 2357.8	0.730	ns
V2 grey matter volume (mm^3^)	13722.9 ± 4837.4	16144.7 ± 8231.2	0.266	ns

Two sample t-test was used to compare between groups for all variables except sex, where chi-squared test was used. *P < 0.05 indicates statistical significance. Data are presented as mean ± standard error. (RNFL: retinal nerve fiber layer; C/D: cup-to-disc ratio; OD: right eye; OS: left eye; ns: not significant).

**Figure 1:**
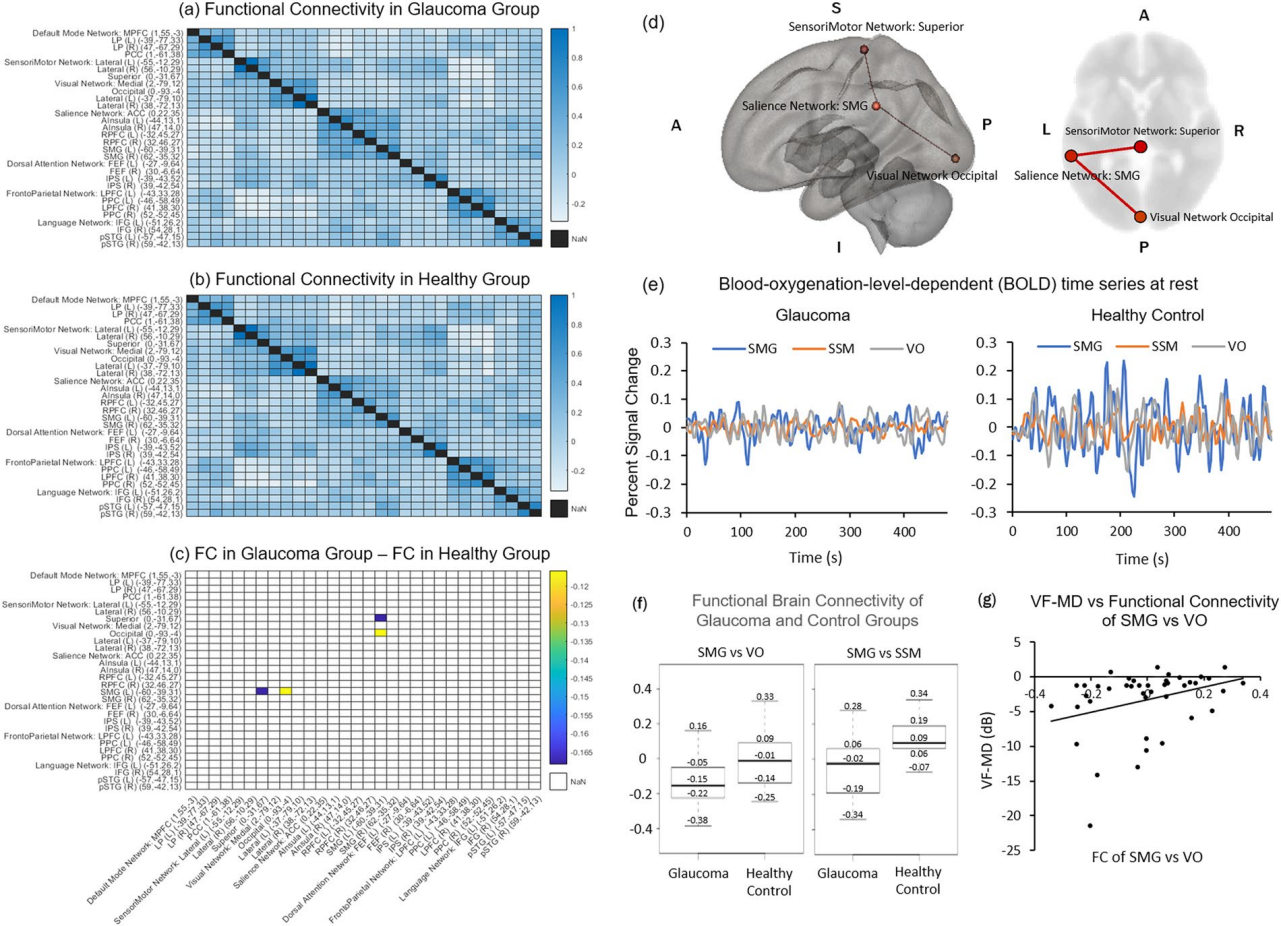
Functional brain connectivity in glaucoma (n = 32) and healthy control (n = 10) subjects. (**a–c)** Heat maps showing correlation coefficients (r) between temporal profiles for each pair of brain regions-of-interest (ROIs) in glaucoma subjects **(a)**, healthy controls **(b)**, and the difference between glaucoma subjects and healthy control (glaucoma – control) **(c)** during task-free functional MRI. The coordinates in brackets indicate the center of ROIs in Montreal Neurological Institute (MNI) space. Details of the seed and target brain regions can be found in Supplementary Information 2. In **(c)**, correlation coefficients are shown only for ROI pairs with significant group differences; (**d**) Three-dimensional sagittal (left) and 2-dimensional axial (right) views of the brain showing the locations of the left supramarginal gyrus (SMG), the visual occipital area (VO), and the superior sensorimotor area (SSM); **(e**) Time series of blood-oxygenation-level-dependent (BOLD) signal percent change for SMG (blue), SSM (orange), and VO (grey) in glaucoma subjects (left) and healthy controls (right); **(f)** Box and whisker plots showing functional connectivity (FC) between SMG and VO areas (left) or between SMG and SSM areas (right) in terms of correlation coefficients (r) for both glaucoma and healthy control subjects. In descending order, values represent: maximum, third quartile, median, first quartile, and minimum. FC between SMG and SSM was greater than zero in healthy controls (p < 0.05) but was not different from zero for glaucoma subjects (p = 0.07). Conversely, FC between SMG and VO was less than zero in glaucoma subjects (p < 0.001) but was not different from zero in healthy controls (p = 0.48); **(g)** Scatter plot showing association between visual field mean deviation (VF-MD) and FC between SMG and VO areas for all subjects.

**Figure 2:**
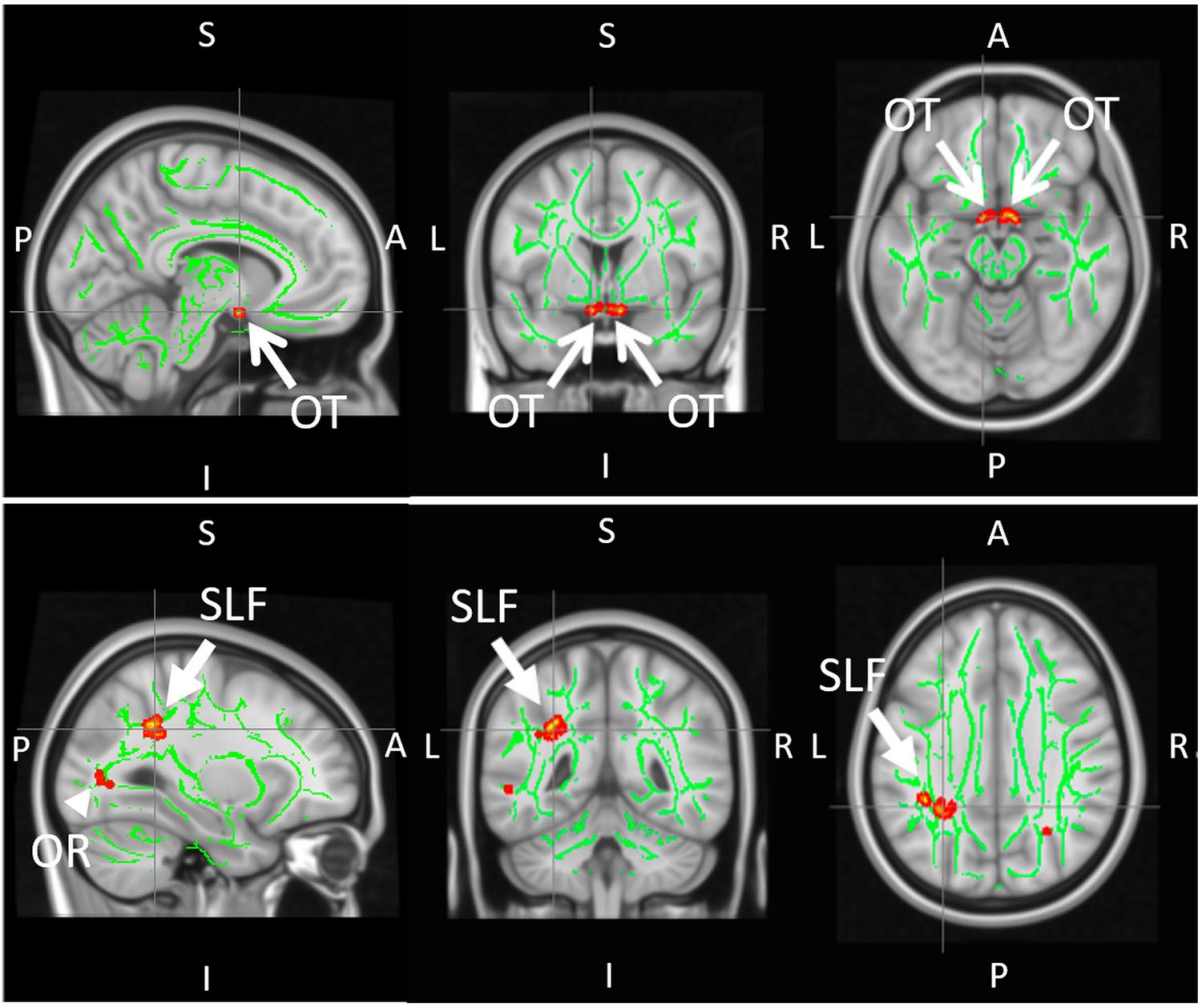
Group comparisons of white matter integrity between glaucoma patients (n = 32) and healthy controls (n = 10) using tract-based spatial statistics (TBSS) of fractional anisotropy (FA) maps in diffusion tensor MRI. Green pixels represent the FA skeletons of major tracts overlaid on the anatomical brain images in grayscale in the sagittal (left column), coronal (middle column) and axial planes (right column). Red/yellow pixels indicate white matter tract regions that had lower FA in glaucoma patients compared to healthy controls (p < 0.05). These regions include the central visual pathway in the optic tracts (OT; open arrows) and optic radiation (OR; arrowhead) (top row), as well as the left superior longitudinal fasciculus (SLF; closed arrows) around the supramarginal gyrus in the non-visual pathway (bottom row). (A: anterior; P: posterior; L: left; R: right; S: superior; I: inferior).

When evaluating functional connectivity in tf-fMRI using all relevant brain regions-of-interest (ROIs) as seeds and targets (see Supplementary Information 2 for details), the left supramarginal gyrus (SMG) had significantly lower functional connectivity with the visual occipital area (VO) (p = 0.04) and the superior sensorimotor area (SSM) (p = 0.04) in glaucoma patients than healthy controls (Fig. 1a–f). VF-MD was also found to be significantly associated with functional connectivity between SMG and VO (r = 0.32, p = 0.04) (Fig. 1g). The SMG is an important brain region for the maintenance of vestibular control during standing balance, active balancing, and caloric vestibular irrigation^30–32^. While visual field loss generally increases the risk of falls in older adults^33^, the decreased ability to integrate multisensory inputs intrinsically in the glaucomatous brain could point to another important central mechanism by which glaucoma patients have increased susceptibility to falls. In this study, the decreased functional connectivity between SMG and visual and sensorimotor regions observed in glaucoma subjects suggests that functional brain reorganization is taking place in these areas. This hypothesis is bolstered by the lower fractional anisotropy observed in the superior longitudinal fasciculus (SLF) around SMG of glaucoma subjects in diffusion tensor MRI (Fig. 2), suggesting compromised structural integrity in the white matter around the SMG in glaucoma subjects. Importantly, the SLF is also known to be coupled to the multimodal brain network that controls vestibular function^34^.

**Figure 3:**
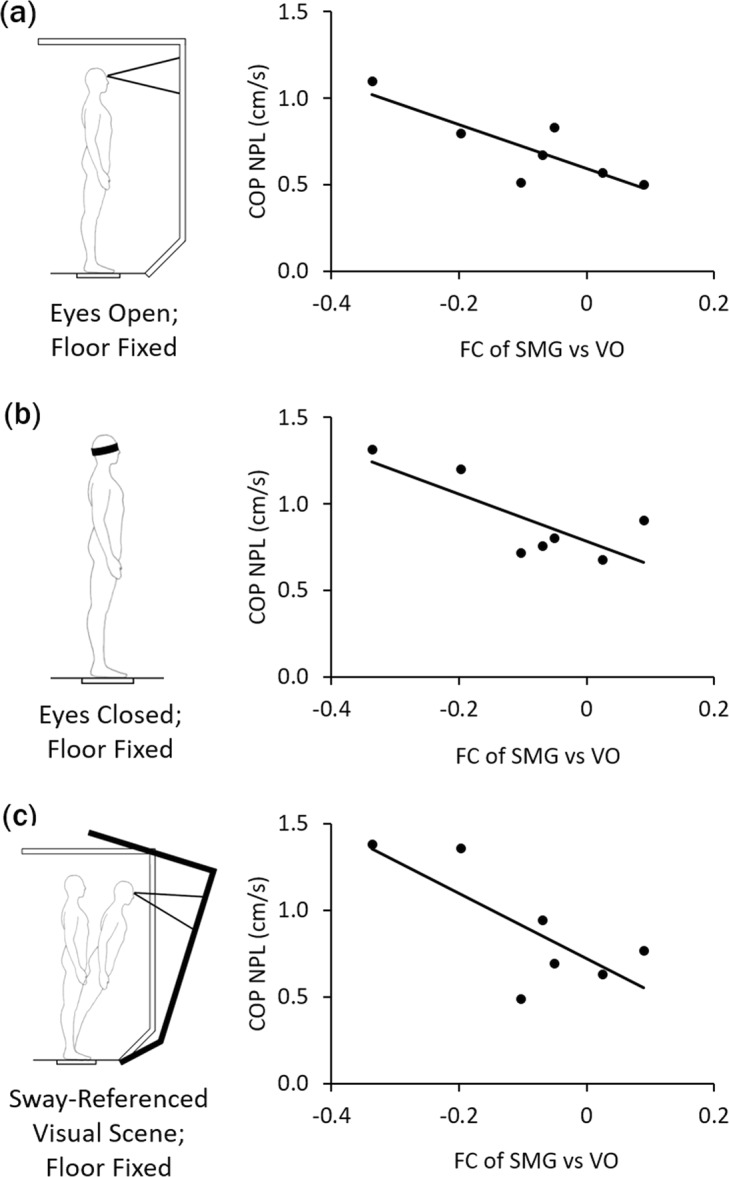
Postural control in glaucoma subjects (n = 7). Scatter plots showing the brain-behavior associations between functional connectivity (FC) of supramarginal gyrus (SMG) versus visual occipital (VO) areas and sway velocity [i.e. normalized path length (NPL) of the center of pressure (COP)] during dynamic posturography under three conditions: **(a)** fixed floor and eyes open (r = −0.83, p < 0.05); **(b)** fixed floor and eyes closed (r = −0.77, p < 0.05); and **(c)** fixed floor in sway-referenced visual environment with altering visual information (r = −0.75, p = 0.05).

Further supporting this hypothesis is the negative association found when comparing sway velocity with functional connectivity between SMG and VO in a subset of 7 glaucoma subjects who underwent dynamic posturography to assess multisensory integration abilities relevant for balance (Fig. 3). This indicates that glaucoma subjects with lower functional connectivity between these two regions had more difficulties in keeping their balance. This trend was observed when visual information was present (eyes open, Fig. 3a, r = −0.83, p = 0.02), absent (eyes closed, Fig. 3b, r = −0.77, p = 0.04) or altered (eyes open with sway-referenced visual scene, Fig. 3c, r = −0.75, p = 0.05) during dynamic posturography, while visual information was absent (eyes closed) during tf-fMRI. Taken together, the altered brain coordination and the corresponding postural control changes in glaucoma patients do not seem necessarily dependent on visual inputs from the eye. The inability of glaucoma patients to effectively integrate multimodal signals has implications for treatment. For instance, physical therapists use different therapeutic approaches for people with balance problems depending upon the amount of central versus peripheral involvements^35–37^. There are also candidate neuroprotective drugs that can be translated from other neurodegenerative diseases to glaucoma. Thus, the current findings can have direct impact on the overall management, rehabilitation and treatment protocols of patients with glaucoma. In terms of the generalizability to other primary blinding disorders, there is some evidence indicating structural and functional brain changes in age-related macular degeneration, diabetic retinopathy, and retinal ischemia, yet these changes are not specific to SMG/SLF or areas that control multimodal integration^38–40^. Future studies are warranted that include other visual disorders of different severity to elucidate the specificity of the current findings to glaucoma.

There are several limitations to the present study. Given the small sample size, the reason for the observed group differences in the left but not right SMG and SLF is unclear. When examining individual groups, the FC between SMG and VO in both hemispheres was significantly less than zero in glaucoma subjects but was apparently indifferent from zero in healthy controls. While some studies reported lateralization of SMG with regard to proprioception^41–43^ and action planning^44–49^, several studies reported bilateral SMG involvements^30–32,50,51^. All in all, larger-scale investigations are required to verify the presence of lateralization observed in this preliminary study. On the other hand, because of the cross-sectional nature of the study, no conclusions could be made about the causality between eye, brain and behavior in glaucoma. Since glaucoma is a chronic disease that progresses slowly over years, longitudinal measurements of a large sample over time are essential to determine the temporal characteristics and causal relationships between biomarkers in the brain and glaucoma with sufficient statistical power. However, our current results are important in the sense that the brain and behavioral changes were already observed in the early stages of glaucoma when clinical ophthalmic assessments showed only small differences between healthy control and glaucoma subjects. Furthermore, as opposed to recent MRI findings showing cortical atrophy in more advanced glaucoma^9,10^, our study did not show apparent difference in macroscopic cortical volumes between glaucoma and healthy control groups using anatomical MRI. This suggests the high sensitivity of tf-fMRI and diffusion tensor MRI in detecting functional and microstructural changes before macroscopic cortical atrophy can be detected in the brains of glaucoma patients. The use of multimodal imaging assessments may allow improved strategies for early and targeted intervention before the disease worsens. The associations between brain changes and visuomotor function also provided important implications of the behavioral relevance underlying the widespread brain changes in glaucoma. If early glaucomatous brain changes and their underlying mechanisms of disease progression and behavioral outcomes can be reliably identified, novel neurotherapeutics targeting parameters beyond intraocular pressure can be useful in the near future, leading to less morbidity from this irreversible but preventable disease.

In summary, the current findings demonstrate the involvement of brain areas responsible for vision and balance in the early stages of glaucoma. Glaucoma patients present reduced structural integrity in the white matter along the central visual pathway and around SMG, as well as reduced functional connectivity between SMG and visual or sensorimotor areas when compared to healthy controls. Furthermore, decreased functional connectivity between SMG and visual brain network may negatively impact postural control. These results have important implications on the central role of visuomotor impairments in glaucoma. They also suggest an involvement of altered brain coordination in the increased risk of falls in glaucoma patients.

## Materials and Methods

### Study approval

All studies have been conducted according to the Declaration of Helsinki principles and were approved by the institutional review board at the University of Pittsburgh, with written informed consent received from participants prior to inclusion in the study.

### MRI Protocol

All MRI experiments were performed on a 3-Tesla Allegra head scanner (Siemens, Erlangen, Germany). Conventional anatomical MRI was first performed covering the whole brain using a 3D MPRAGE pulse sequence with the following parameters: repetition time (TR) = 1.4 s, echo time (TE) = 2.5 ms, inversion time (TI) = 800 ms, flip angle = 8°, field of view = 25.6 × 25.6 × 17.6 cm^3^, 256 × 256 imaging matrix in-plane, and 176 contagious sagittal slices. Blood-oxygen-level-dependent (BOLD) images were then collected for tf-fMRI using a single-shot echo-planar-imaging (EPI) pulse sequence with the following parameters: TR = 2 s, TE = 26 ms, field-of-view = 20.5 × 20.5 cm^2^, 104 × 104 imaging matrix, and 28 contiguous 3 mm thick axial slices. The total duration of tf-fMRI was 8 min with eyes closed at rest. Diffusion tensor MRI was acquired covering the whole brain using spin-echo diffusion-weighted EPI sequences with the following parameters: 12 diffusion gradient directions at diffusion weighting factor (b) = 850 s/mm^2^ and one b = 0 s/mm^2^ (b_0_), TR = 5.2 s, TE = 80 ms, field-of-view = 20.5 × 20.5 cm^2^, 104 × 104 imaging matrix, and 38 axial slices at 3 mm thickness. We used landmarks based on anatomical MRI and the human brain atlas to ensure consistent localization of tf-fMRI and diffusion tensor MRI measurements across subjects.

### Standing balance assessment

The standing balance test used dynamic posturography on an Equitest posture platform (Neurocom, Inc). This platform is capable of sway-referencing the visual environment to reduce balance-related visual cues via rotating the visual scene in direct proportion to an individual’s sway magnitude in the anterior-posterior direction^52^. The platform records ground reaction forces under the feet during standing and underfoot center of pressure (COP). Participants wore a safety harness that would prevent hitting the floor in the event of a balance loss. During balance testing, participants were instructed to stand as still as possible without locking their knees. Participants were assessed in either eyes closed or eyes open with or without sway-referenced visual scene. These postural conditions are parts of the well-established balance testing paradigm, namely the Sensory Organization Test, which has been used and validated in healthy and clinical populations^53^. An adapted version of the Sensory Organization Test was used, with each postural condition lasting 3 minutes.

### Data processing and analysis

To ensure robust and unbiased results, grouping identifiers were blinded to our researchers until statistical tests were performed. From anatomical MRI, optic nerve volumes were manually estimated using ImageJ (https://imagej.nih.gov/ij/), whereas grey matter volumes in primary and secondary visual cortices were derived from voxel-based morphometry using FreeSurfer (http://surfer.nmr.mgh.harvard.edu). All images were normalized into standard Montreal Neurological Institute (MNI) space and segmented before volume estimation for each subject. For tf-fMRI, the Functional Connectivity (CONN) toolbox was used for data preprocessing and ROI analyses (https://www.nitrc.org/projects/conn/). For preprocessing, realignment, unwarping, slice-timing correction, co-registration with structural images, spatial normalization into the standard MNI space, and smoothing were performed. A component based noise correction method (CompCor) was used to identify principal components and specify nuisance regressors pertaining to white mater and cerebrospinal fluid for each subject. The preprocessed images were then band-passed filtered over 0.008 to 0.09 Hz to extract low frequency fluctuations pertinent to resting-state functional connectivity. ROI regions were defined by 10 mm spheres centered at the coordinates specified by the Automated Anatomical Labeling and the Harvard-Oxford Atlas. The exact ROIs used are detailed in Supplementary Information 2. CONN performed seed-based analysis by calculating the temporal correlation between the defined set of seed voxels and all other voxels. From these first level analyses, second level analyses were calculated by performing t-tests, which identified regions that had significant changes in functional connectivity between the two experimental groups. False discover rate corrections were applied in order to correct for multiple comparisons.

For diffusion tensor MRI, preprocessing of the native diffusion weighted images was performed with Freesurfer to correct for eddy currents and motion artifacts and for tensor regression. Non-linear registration into the MNI152 standard space was then applied to the fractional anisotropy maps to perform voxelwise comparisons using tract-based spatial statistics (TBSS) via the FMRIB Software Library (FSL) (http://www.fmrib.ox.ac.uk/fsl) and custom-written software.

For standing balance assessment, the primary outcome variable was based on the COP data. Specifically, the anterior-posterior average sway velocity was assessed using the COP’s time-normalized path length (COP-NPL)^54^. The COP-NPL is proportional to sway velocity and is thus an assessment of the degree of “control” or level of effort generated by the postural control system to maintain balance.

Pearson product-moment correlation test was used to evaluate the association between functional connectivity and swing speed for different postural conditions. Correlation tests were also performed between clinical and MRI measures. Two sample t-test was used to compare between groups for all clinical, volumetric MRI and demographic variables except sex, where chi-squared test was used. Results were considered significant when p < 0.05.

## Supplementary information

Supplementary Information
