# Supplementary material for: Widespread brain reorganization perturbs visuomotor coordination in early glaucoma
Source: Sci Rep. 2019 Oct 2;9:14168. doi: 10.1038/s41598-019-50793-x (PMC6775162; doi:10.1038/s41598-019-50793-x)
Supplement: Supplementary file 1 — Supplementary Information [file 41598_2019_50793_MOESM1_ESM.pdf]

Supplementary Information

Title: Widespread brain reorganization perturbs visuomotor coordination in early glaucoma

Author list: Vivek Trivedi, Ji Won Bang, Carlos Parra, Max K. Colbert, Caitlin O'Connell, Ahmel Arshad, Muneeb A. Faiq, Ian P. Conner, Mark S. Redfern, Gadi Wollstein, Joel S. Schuman, Rakie Cham, and Kevin C. Chan[#]

[#]Corresponding author

Supplementary Information 1: In this observational, cross-sectional study, 32 glaucoma subjects [age= 62.5±1.5 (mean ± standard error); 40.6% male] and 10 healthy controls [age= 64.5±2.9; 30.0% male] were recruited between Jan 2013 and Dec 2017 at the University of Pittsburgh. Detailed inclusion and exclusion criteria can be found below:

Inclusion Criteria:

1. Male or female, ages 18 years and older.

2. Patients that were defined clinically as glaucoma.

3. All healthy control subjects must not have any known neurological disorders or any medical condition that can possibly lead to emergency medical care.

4. Able to read (or have read to him or her), understand, and sign the Informed Consent form.

5. Height less than or equal to 6'4".

6. Weight less than or equal to 250 lbs.

7. Ability to walk and stand independently.

Exclusion Criteria:

1. Presence of any foreign metal in the body with the exception of dental fillings will need to be prescreened

by radiology prior to signing the informed consent and enrollment.

2. Pregnant or breastfeeding. Women of childbearing age will undergo a urine pregnancy screen prior to signing the informed consent and enrollment.

3. Any medical condition that would interfere with the ability to lie supine in the MRI scanner or to perform the limited study tasks in the MRI scanner.

4. Implanted electrical medical devices such as pacemakers.

5. Implanted metallic or ferromagnetic objects (aneurysm clip, ear implant, IUD, shrapnel or metallic fragments in or on the body or eyes, neurostimulators, or other metal devices).

6. Any ocular pathology other than glaucoma.

7. Noticeable anxiety and claustrophobia that would prevent functional neuroimaging.

8. Obesity preventing placement in MRI scanner.

9. Musculoskeletal, cardiovascular or neurological condition (if not controlled with medication and/or if it limits ability to stand for 2 hours without pain or discomfort).

10. History of weight bearing joint surgery or arthroplasty (if done less than 1 year ago and/or if it limits ability to stand for 2 hours without pain or discomfort).

11. Arthropathy (such as severe arthritis) or pain that limits ability to stand for 2 hours without pain or discomfort.

12. Taking any medications for your vestibular system.

13. Taking any CNS depressant drugs (benzodiazepines or barbiturates).

14. Self-reported balance problems.

15. Use of gait assistive devices, such as a cane or walker.

Supplementary Information 2: List of regions of interest (ROIs) used in functional connectivity MRI analysis with coordinates:

networks.DefaultMode.Medial Prefrontal Cortex (1,55,-3)'

networks.DefaultMode.Lateral Parietal Cortex (L) (-39,-77,33)'

networks.DefaultMode.Lateral Parietal Cortex (R) (47,-67,29)'

networks.DefaultMode.Posterior Cingulate Cortex (1,-61,38)'

networks.SensoriMotor.Lateral (L) (-55,-12,29)'

networks.SensoriMotor.Lateral (R) (56,-10,29)'

networks.SensoriMotor.Superior (0,-31,67)'

networks.Visual.Medial (2,-79,12)'

networks.Visual.Occipital (0,-93,-4)'

networks.Visual.Lateral (L) (-37,-79,10)'

networks.Visual.Lateral (R) (38,-72,13)'

networks.Salience.Anterior Cingulate Cortex (0,22,35)'

networks.Salience.Anterior Insula (L) (-44,13,1)'

networks.Salience.Anterior Insula (R) (47,14,0)'

networks.Salience.Rostral PreFrontal Cortex (L) (-32,45,27)'

networks.Salience.Rostral PreFrontal Cortex (R) (32,46,27)'

networks.Salience.SupraMarginal Gyrus (L) (-60,-39,31)'

networks.Salience.SupraMarginal Gyrus (R) (62,-35,32)'

networks.DorsalAttention.Frontal Eye Field (L) (-27,-9,64)'

networks.DorsalAttention.Frontal Eye Field (R) (30,-6,64)'

networks.DorsalAttention.IntraParietal Sulcus (L) (-39,-43,52)'

networks.DorsalAttention.IntraParietal Sulcus (R) (39,-42,54)'

networks.FrontoParietal.Lateral PreFrontal Cortex (L) (-43,33,28)'

networks.FrontoParietal.Posterior Parietal Cortex (L) (-46,-58,49)'

networks.FrontoParietal.Lateral PreFrontal Cortex (R) (41,38,30)'

networks.FrontoParietal.Posterior Parietal Cortex (R) (52,-52,45)'

networks.Language.Inferior Frontal Gyrus (L) (-51,26,2)'

networks.Language.Inferior Frontal Gyrus (R) (54,28,1)'

networks.Language.Posterior Superior Temporal Gyrus (L) (-57,-47,15)'

networks.Language.Posterior Superior Temporal Gyrus (R) (59,-42, 13)'
